# Obituary

**Published:** 2008

**Figure:**
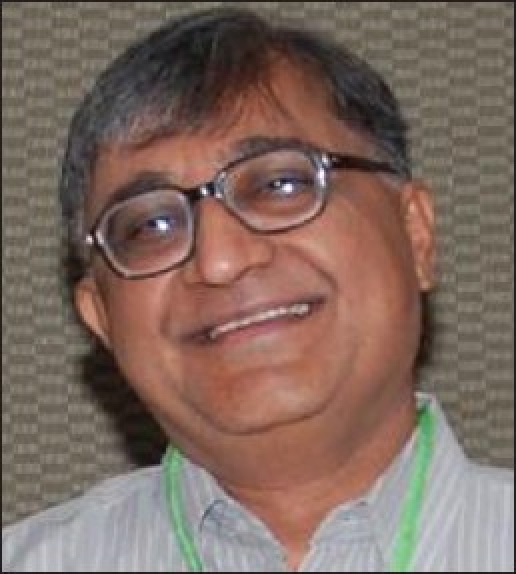
Dr. Kamlesh Kumar Sharma, Hon. Secretary, Indian Association of Pediatric Surgeons (11^th^ March 1957 - 17^th^ August 2008)

The Journal of Indian Association of Pediatric Surgeons (JIAPS) deeply regrets the sudden passing away of one of our esteemed editorial board member and senior colleague Dr. Kamlesh K Sharma, Associate Professor of Pediatric Surgery at the Dr. S. N. Medical College, Jodhpur, Rajasthan. He completed his MCh in 1988 from the SMS Medical College, Jaipur and commenced his career in Jodhpur in 1991. By single handed work over 17 years he developed Pediatric Surgery as a superspecialty in Western Rajasthan. He had an excellent scientific temper and an enquiring mind. He had to his credit the identification, naming and classification of a disease – acquired gastric outlet obstruction in children which had been christened ‘Jodhpur disease’. This unique contribution was applauded by the international community of Pediatric Surgeons and was published in the prestigious Journal of Pediatric Surgery in Oct 2008. A popular speaker he was a Visiting Professor in many universities in Europe. This year he was scheduled to address the Mediterranean Association of Pediatric Surgeons in Tunis and to give a talk at the Bochum University in Germany. He was chosen by the Govt of India for the ‘Rajiv Gandhi Award’ which was presented to him in New Delhi in August 2007. He represented the North Zone as an Executive Member in the IAPS Secretariat. 

Gentle by nature, Dr. Sharma valued friendships and would often go out of the way to make things easy for his colleagues and friends. His students fondly remember his support and guidance during training. A proof of his acceptance all over India was his election in 2007 to the prestigious post of ‘Secretary – Indian Association of Pediatric Surgeons’. He was full of ideas and was tirelessly working to make the forthcoming annual conference at Guwahati a resounding success on the scientific front. Honest and fair to a fault, Dr. Sharma made sure that every scientific paper submitted was screened and reviewed without bias and graded. The news of his death was received with disbelief and messages of condolence poured into the association website from home and abroad. 

The post of Secretary often takes a toll on family and work. Maybe it was preoccupation with the onerous task which made him step inadvertently into the empty well of a lift. The Chinese believe that at 50 years one is at the peak of his career. It is very unfortunate that this gentle soul was so cruelly snatched from our midst at a time when he had so much more to offer. 

His wife Mrs. Sudha Sharma was his ardent companion and shared his love of friends. She actively helped him in the preparations for the Guwahati conference. His two sons Parikshit and Anirudh have done him proud. The eldest is a graduate engineer in computer science and is pursuing his Master’s in the United States. The second son is training to be a Dental Surgeon at Manipal University.

The JIAPS salutes the family for being so brave during the crisis and for no leaving no stone unturned in his treatment. Dr Kamlesh K Sharma will never be forgotten by the JIAPS. A condolence meeting was held on 22^nd^ August in Bangalore at the NIMHANS Convention Center in Bangalore and over a hundred pediatric surgeons expressed their shock and anguish.

The Association prays to the Almighty to provide solace and comfort to the bereaved family and for peace to the departed soul.

